# Variation in seizure risk increases from antiseizure medication withdrawal among patients with well‐controlled epilepsy: A pooled analysis

**DOI:** 10.1002/epi4.12880

**Published:** 2023-12-16

**Authors:** Samuel W. Terman, Geertruida Slinger, Adriana Koek, Jeremy Skvarce, Mellanie V. Springer, Julie M. Ziobro, James F. Burke, Willem M. Otte, Roland D. Thijs, Morten I. Lossius, Anthony G. Marson, Laura J. Bonnett, Kees P. J. Braun

**Affiliations:** ^1^ Department of Neurology University of Michigan Ann Arbor Michigan USA; ^2^ Department of Child Neurology, UMC Utrecht Brain Center, Wilhelmina Children's Hospital University Medical Center Utrecht and Utrecht University Utrecht The Netherlands; ^3^ Department of Neurology University of California San Francisco San Fransisco California USA; ^4^ University of Michigan Medical School Ann Arbor Michigan USA; ^5^ Department of Pediatrics University of Michigan Ann Arbor Michigan USA; ^6^ Department of Neurology The Ohio State University Columbus Ohio USA; ^7^ Stichting Epilepsie Instellingen Nederland (SEIN) Heemstede The Netherlands; ^8^ Department of Neurology Leiden University Medical Centre (LUMC) Leiden The Netherlands; ^9^ Queen Square Institute of Neurology University College London London UK; ^10^ Oslo University Hospital National Center for Epilepsy Oslo Norway; ^11^ Institute of Clinical Medicine, University of Oslo Oslo Norway; ^12^ Department of Pharmacology and Therapeutics University of Liverpool Liverpool UK; ^13^ Department of Health Data Science University of Liverpool Liverpool UK

**Keywords:** antiseizure medications, discontinuation, epilepsy, risk prediction

## Abstract

**Objective:**

Guidelines suggest considering antiseizure medication (ASM) discontinuation in seizure‐free patients with epilepsy. Past work has poorly explored how discontinuation effects vary between patients. We evaluated (1) what factors modify the influence of discontinuation on seizure risk; and (2) the range of seizure risk increase due to discontinuation across low‐ versus high‐risk patients.

**Methods:**

We pooled three datasets including seizure‐free patients who did and did not discontinue ASMs. We conducted time‐to‐first‐seizure analyses. First, we evaluated what individual patient factors modified the relative effect of ASM discontinuation on seizure risk via interaction terms. Then, we assessed the distribution of 2‐year risk increase as predicted by our adjusted logistic regressions.

**Results:**

We included 1626 patients, of whom 678 (42%) planned to discontinue all ASMs. The mean predicted 2‐year seizure risk was 43% [95% confidence interval (CI) 39%–46%] for discontinuation versus 21% (95% CI 19%–24%) for continuation. The mean 2‐year absolute seizure risk increase was 21% (95% CI 18%–26%). No individual interaction term was significant after correcting for multiple comparisons. The median [interquartile range (IQR)] risk increase across patients was 19% (IQR 14%–24%; range 7%–37%). Results were unchanged when restricting analyses to only the two RCTs.

**Significance:**

No single patient factor significantly modified the influence of discontinuation on seizure risk, although we captured how absolute risk increases change for patients that are at low versus high risk. Patients should likely continue ASMs if even a 7% 2‐year increase in the chance of any more seizures would be too much and should likely discontinue ASMs if even a 37% risk increase would be too little. In between these extremes, individualized risk calculation and a careful understanding of patient preferences are critical. Future work will further develop a two‐armed individualized seizure risk calculator and contextualize seizure risk thresholds below which to consider discontinuation.

**Plain Language Summary:**

Understanding how much antiseizure medications (ASMs) decrease seizure risk is an important part of determining which patients with epilepsy should be treated, especially for patients who have not had a seizure in a while. We found that there was a wide range in the amount that ASM discontinuation increases seizure risk—between 7% and 37%. We found that no single patient factor modified that amount. Understanding what a patient's seizure risk might be if they discontinued versus continued ASM treatment is critical to making informed decisions about whether the benefit of treatment outweighs the downsides.

**Key points**

- We pooled three datasets containing patients with well‐controlled epilepsy who continued versus discontinued antiseizure medications.
- No individual patient characteristic significantly modified the odds ratio of having a seizure from discontinuation.
- The 2‐year absolute seizure risk increase from discontinuation ranged from 7% (in the lowest‐risk patients) to 37% (in the highest‐risk patients), with most patients falling in between.
- Our work provides a basis for shared decision‐making and will lead to future work developing an individualized calculator and contextualizing seizure risk according to patient preferences.

## 1 INTRODUCTION

Over 50 million people have epilepsy worldwide.^1^ Fortunately, two‐thirds of patients become seizure free on antiseizure medications (ASMs).^2^ For this group, a central question is whether ASMs are necessary indefinitely. ASMs reduce morbidity and mortality by reducing seizures.^3^ However, ASMs may cause side effects^4^ that reduce quality of life.^5^ Seizure risk declines with increasing seizure‐free intervals such that the absolute benefit of continued ASMs reduces over time.^6^ Accordingly, guidelines have endorsed considering ASM discontinuation for patients with acceptably low post‐withdrawal seizure risk after detailed risk‐benefit discussions.^7^, ^8^, ^9^

Two randomized trials have evaluated the average seizure risk increase from discontinuation.^10^, ^11^, ^12^ However, many patients will almost certainly derive greater or lesser than average benefit. Prior work has incompletely described the degree to which effects vary between patients. A “positive” trial (*p* < 0.05) suggests that, on average, ASMs reduce the chance of seizure recurrence.^10^ However, this does not mean that all individuals within any group benefit or benefit equally from treatment, or does a “negative” trial (group difference *p*‐value > 0.05^11^) mean that no patients benefit. Calculating an individual's risk difference (comparing discontinuation vs. continuation) is needed to achieve precision medicine and personalize care, beyond what can be understood from group averages.

Additionally, an online calculator exists computing individualized post‐discontinuation seizure relapse risks.^13^, ^14^ However, as above, a key piece is missing: what would the patients risk have been if they continued ASMs? Calculating only post‐discontinuation risk without an explicit comparison to continuation fails to distinguish critically different scenarios, such as patients who would be high risk regardless of treatment (little benefit from treatment), versus patients who would be low risk on treatment but high risk off treatment (high benefit from treatment). Yet, prior work has largely paid attention to calculating only individualized post‐discontinuation risks (“what factors suggest a higher or lower risk patient”, ie, risk factors) without comparison to individualized continuation risks (“what factors suggest a more or less effective treatment”, ie, effect modifiers).

The 1991 MRC study did develop a two‐armed individualized risk score under discontinuation versus continuation. However, that represented a single study, lacked potentially important variables (eg, syndrome, number of past seizures, and prior status epilepticus), and did not explicitly lay out the distribution of anticipated treatment effects.^15^ A better understanding of how much discontinuation may increase seizure risk across patients would foster more detailed counseling.

We asked: (1) Does discontinuation increase the odds of another seizure more so in some groups than others? (2) What is the possible range of seizure risk increases due to discontinuation, for patients at different levels of post‐discontinuation risk? This latter question would inform the possible minimum and maximum effect of withdrawal on seizure risk from a population perspective. For example, how much might withdrawal increase for a low‐risk patient? How much for a high‐risk patient? And where do most patients fall along this continuum?

## 2 METHODS

### 2.1 Study design; datasets

We pooled data from the three available studies that contain any appreciable number of seizure‐free patients including adults, some who planned to discontinue and others planning to continue ASMs. This was per our systematic search strategy described in previous meta‐analyses^6^, ^13^ extended to January 1, 2022.

1. Medical Research Council (MRC) RCT^10^: Adults and children with epilepsy who were at least 2 years seizure free were randomized to discontinue (decrementing doses every ~4 weeks until off) versus continue ASMs, across 40 centers in the United Kingdom. Five percent of the withdrawal group did not withdraw and 35% of the continuation group did not continue, but this resulted in negligible bias.^16^
2. The Akershus RCT (Lossius et al.^11^): Adults with epilepsy, excluding high‐risk features (eg, juvenile myoclonic epilepsy), who were seizure free at least 2 years were randomized to discontinue (by approximately 20% every other week until off) versus continue ASMs, from one hospital's catchment area in Norway. Note that 146/149 (98%) of analyzed patients in the Lossius study followed their assigned protocol.
3. Real‐world retrospective multi‐institution observational cohort^17^: All patients at least 1 years old at the time of their first outpatient visit when they were seizure free for at least 1 year, excluding specific high‐risk features (eg, juvenile myoclonic epilepsy), were followed over maximally 7 years, from three health systems (University of Michigan; Stichting Epilepsie Instellingen Nederland; and Wilhelmina Childrens Hospital). Despite lack of randomization, this study provided a key methodological advantage over other withdrawal cohorts: this cohort followed all patients regardless of whether they withdrew ASMs and recorded the day on which it was decided to withdraw ASMs as documented in the electronic medical record. Capturing both “pre” versus “post” discontinuation periods provided within‐subject data, while adjusting for seizure‐free times, thus reducing confounding or selection bias otherwise inherent to cohorts following only post‐discontinuation patients. Still, in sensitivity analyses, we restricted to only the above two RCTs.

### 2.2 Statistical analysis

#### 2.2.1 Evaluating the average effect of discontinuation: “Base model”

We produced average cumulative seizure incidence curves for discontinuation versus continuation with a time‐to‐event discrete logistic regression model (with a cubic term for time) via the cumulative product method akin to Kaplan‐Meier curve methodology (multiplying each months seizure‐free probability times the previous months value).^18^, ^19^ This enabled us to avoid the proportional hazards assumption, which was violated in MRC.^16^ We standardized over confounders listed in Table S1. For RCTs, the main predictor was randomized arm throughout. For the observational cohort, the main predictor started out as “continuation,” which switched to “discontinuation” in the month when they decided to discontinue, if ever, to correctly attribute pre‐ versus post‐discontinuation time. Multiple imputations addressed missing data.^20^, ^21^, ^22^, ^23^

#### 2.2.2 Evaluating what factors modify the effect of discontinuation: “Individual Interactions Model”

We evaluated what patient factors modified the odds ratio of discontinuation on seizure risk by adding interaction terms to the base model, between discontinuation and each variable listed in Table S1. Note that main effects for each risk factor in a model (ie, Table S3) evaluate what variables suggest a higher‐ or lower‐risk patient. In contrast, interaction terms between risk factors and discontinuation in a model (ie, *p*‐values in Table 1) evaluate for whom treatment is most effective.

**TABLE 1:** Patient population at the start of follow‐up.

	No./*N* (%) or median (IQR; *N*)	Adjusted *p*‐value for interaction with discontinuation	
		Corrected for multiple comparisons?^a^	
		No	Yes
Study			
MRC	1013/1626 (62%)	Reference	Reference
Chart review	464/1626 (29%)	0.79	0.92
Lossius^b^	149/1626 (9%)	0.23	0.96
Age at epilepsy diagnosis category^c^			
<11	588/1614 (36%)	Reference	Reference
11–17	395/1614 (24%)	0.60	0.83
18+	631/1614 (39%)	0.33	>0.99
Age at epilepsy diagnosis^c^	14 (7–23; 1465)		
Years seizure‐free category^c^			
<2	206/1625 (13%)	—	—
2 to <3	523/1625 (32%)	—	—
3 to <5	365/1625 (22%)	—	—
5+	531/1625 (33%)	—	—
Number of ASMs			
1	1253/1626 (77%)	Reference	Reference
2	307/1626 (19%)	0.83	0.86
3+	66/1626 (4%)	0.74	0.93
Seizures impairing awareness	857/1625 (53%)	**0.02**	0.50
Age at start of follow‐up	29 (18–45; 1626)	**0.05**	0.63
Epileptiform EEG	454/1625 (28%)	0.14	0.94
Febrile seizures	142/1473 (10%)	0.15	0.94
Prior status epilepticus	105/451 (23%)	0.22	0.96
Female	816/1626 (50%)	0.35	>0.99
>9 lifetime seizures	366/456 (80%)	0.39	>0.99
Self‐limited syndrome	17/458 (4%)	0.41	0.94
Developmental delay	235/1625 (14%)	0.42	0.92
Prior brain surgery^d^	49/1477 (3%)	0.44	0.85
Older‐generation ASM^e^	1411/1626 (87%)	0.44	0.85
Newer‐generation ASM	330/1626 (20%)	—	—
Motor seizures^40^	1420/1625 (87%)	0.53	0.90
Years of seizures before remission	5 (1–14; 1465)	0.54^f^	0.88
Years of seizure free	3 (2–5; 1477)	0.56	0.88
Family history of seizures	257/1475 (17%)	0.60	0.83
Abnormal neurological exam	107/1162 (9%)	0.71	0.93
Prior discontinuation attempt	262/1519 (17%)	0.81	0.90
Focal epilepsy	793/1617 (49%)	0.83	0.85
Structural etiology	298/1626 (18%)	0.85	0.85

Abbreviation: ASMs, antiseizure medications.

^a^ Uncorrected *p*‐values represent interaction terms between each listed variable and discontinuation, from a discrete‐time logistic regression including main effects and interactions for all listed variables. *p*‐Values were corrected upward by setting the false discovery rate to 5%.^24^ Note that, per Table S1, three variables were present only in the retrospective cohort, thus displayed *p*‐values for those variables (self‐limited syndrome, more than nine seizures, status epilepticus) are from a model fit only on the retrospective cohort to avoid imputing such a large fraction. Conclusions were similar when assessing *p*‐values for those variables imputing the datasets not containing those variables (self‐limited syndrome: uncorrected *p* = 0.41, corrected *p* = 0.78; more than nine seizures: uncorrected *p* = 0.39, corrected *p* = 0.82; status epilepticus: uncorrected *p* = 0.06, corrected *p* = 0.48).

^b^ Lossius et al. randomized 160 patients but due to exclusions or protocol violations analyzed only 149.

^c^ Age at epilepsy diagnosis and years since last seizure were both entered into the model as categorical variables, given Lossius et al. recorded only categories rather than the underlying continuous value, and categorizing also reduced reliance upon linear assumptions.

^d^ Note that no patient in this dataset had brain surgery for drug refractory epilepsy. The listed surgeries were for other primary indications to treat the underlying condition. Also, note that we list prior brain surgery in this Table to describe our population. However, this variable was completely collinear with other variables (eg, >9 lifetime seizures) and thus this variable had to be removed from the base model to achieve convergence. Hence, prior brain surgery does not appear in Table S3.

^e^ Older generation: carbamazepine, diazepam, mogadon, phenobarbital, phenytoin, primidone, prominal, sulthiame, tridione, valproate, and ethosuximide. All others were considered newer generation. Given we entered older generation into our models, we did not also enter new generation.

^f^ The *p*‐value displayed is for log (years of seizures before remission), given this skewed variable required transformation to achieve convergence in our imputation models.

Because exploratory subgroup analyses risk finding false positives, we used the Benjamini‐Hochberg method setting the false discovery rate to 5%.^24^

#### 2.2.3 Evaluating the distribution of seizure risk increases due to discontinuation across patients (“Risk Interaction Model”)

We then asked, “What was the absolute seizure risk increase due to discontinuation amongst the lowest‐risk patients? What about for the highest‐ risk patients? Where do most patients fall along this spectrum?”

One typical approach to evaluating whether treatment affects groups differently, like above in the individual interactions model, involves displaying odds ratios for groups “one‐at‐a‐time” (eg, is treatment more effective for males vs. females, for normal EEG vs. abnormal EEG, etc.).^25^ However, in addition to possible false positives due to multiple comparisons,^26^ this conventional approach may also miss true differences—comparing groups based on only one factor at time does not represent the full range of differences between patients.^26^

Therefore, our primary analysis used an alternative approach called risk‐based modeling (“Risk Interaction Model”).^26^, ^27^, ^28^ Risk‐based modeling evaluates how the effect of treatment varies not according to just any single group at a time (individual interactions), but rather according to a patient's overall multivariable risk (risk interaction). In other words, the question is no longer, for example, “How does the effect of discontinuation on seizures differ for males versus females?” but rather the more encompassing question, “How does the effect of discontinuation on seizures differ for a high‐ versus low‐risk patient?”^29^

To do so, we calculated each patient's predicted 2‐year post‐discontinuation seizure risk from the base model. To calculate this quantity, we set each patient's treatment variable to “discontinuation” throughout, regardless of their actual treatment, and then took the cumulative product of each patient's seizure‐free probability each month up until 2 years.^30^ We then included an interaction between that quantity and whether they actually discontinued to allow for the possibility that the seizure odds ratio due to discontinuation may differ for higher‐ versus lower‐risk patients.

We displayed results in two complementary ways. First, to display detailed cumulative seizure risk information across time, we calculated predicted survival curves at three hypothetical levels of 2‐year seizure risk, for discontinuation versus continuation. We chose levels to explore most of the range of our population's risk distribution: 20% 2‐year post‐discontinuation seizure risk to represent “low risk” (the 11th percentile of 2‐year risks as predicted from the base model), 50% (the 76th percentile), and 80% (the 99th percentile). Second, we displayed discontinuation versus continuation seizure risks within 2 years, across the full range of seizure risks. We did so to display detailed seizure risks at a single point in time, across all possible risk levels.

We performed two sensitivity analyses. First, we repeated our main analysis except using 2‐year discontinuation risks as calculated from the externally derived Lamberink model.^13^ Second, we repeated our main analyses limited to only randomized datasets.

Data were analyzed using SAS version 9.4 (Cary, NC) and Stata version 17.0 (College Station, TX).

## 3 RESULTS

### 3.1 Population description

We included 1626 patients [MRC: 1013 (62%); chart review: 464 (29%); Lossius: 149 (9%)], with a total of 4962 person‐years of follow‐up. Table 1 describes the overall population's characteristics. Table S2 describes each study's characteristics.

There were 678 (42%) patients who planned to discontinue at any point, either due to randomization [MRC: 510/1103 (50%); Lossius: 72/149 (48%)] or else as planned at office visits during follow‐up [chart review: 96/464 (21%)].

For patients with any seizure during follow‐up, the first seizure occurred at a median of 12 months (min: <1; IQR 5–25; max 83). The chance of having any seizure at any point during follow‐up was 41% (281/678) for patients who planned to discontinue at any point versus 33% (313/948) for patients who planned to continue.

For patients without any seizure during follow‐up, follow‐up lasted a median of 49 months (min: <1; IQR 22–68; max 93). Median follow‐up for patients without any seizure during follow‐up was 49 months (IQR 25–66) for patients who planned to discontinue at any point versus 50 months (IQR 17–68) for patients who planned to continue throughout.

### 3.2 Evaluating the average effect of discontinuation on seizure risk (“Base Model”)

Table S3 displays the base model's coefficients. For example, discontinuation, shorter years of seizure free, more years of seizures before remission, and non‐Lossius studies were correlated with greater odds of a seizure (*p* < 0.05).

**FIGURE 1:**
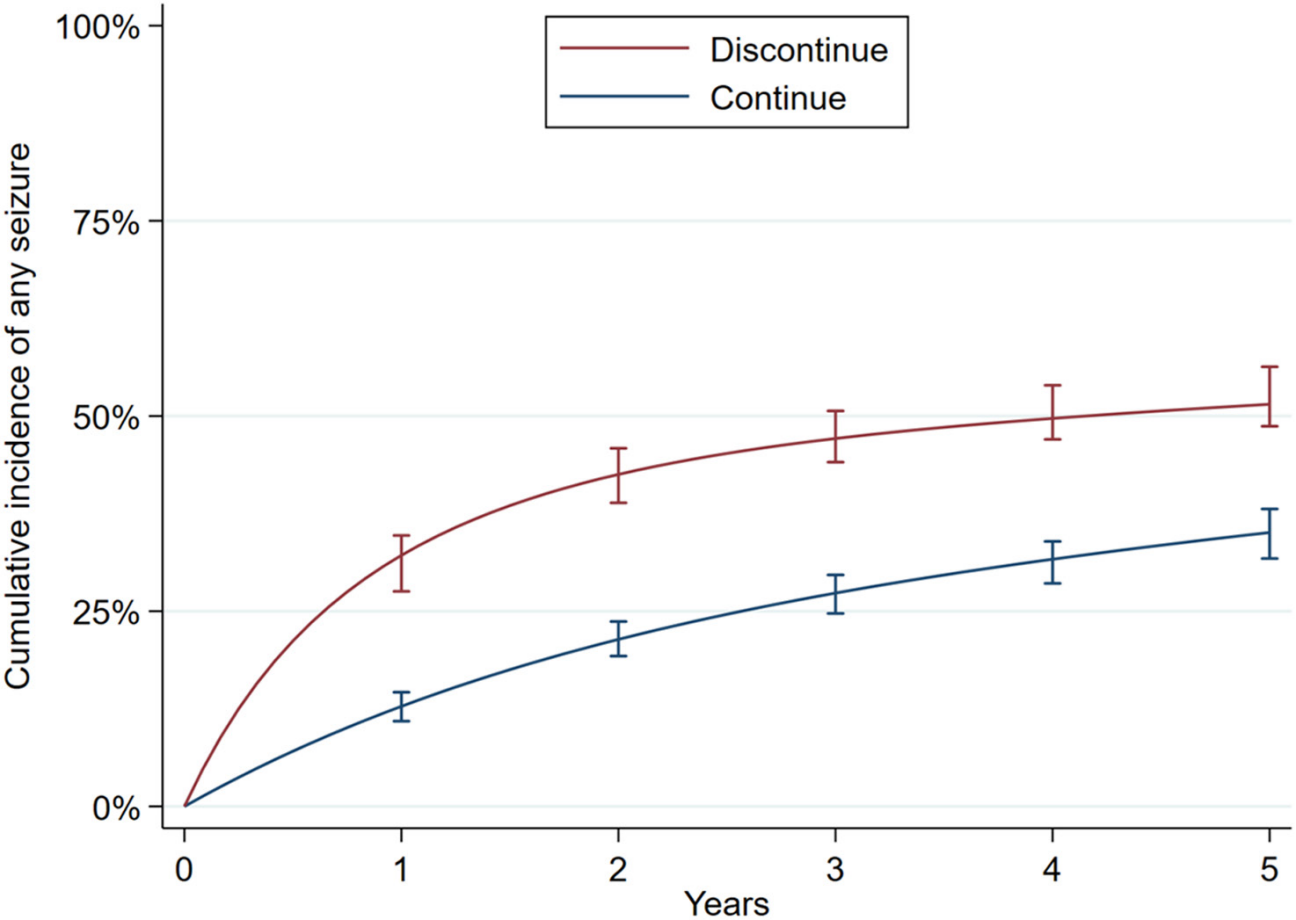
Adjusted cumulative seizure incidence over time for discontinuation versus continuation. Averages, from the base model. Error bars represent bootstrapped 95% confidence intervals.

Figure 1 displays standardized cumulative incidence functions for discontinuation versus continuation. One‐year seizure risk was 32% (95% CI 28%–35%) for discontinuation versus 13% (95% CI 11%–15%) for continuation. The absolute 1‐year risk increase was thus 19% (95% CI 16%–23%). The 2‐year seizure risk was 43% (95% CI 39%–46%) for discontinuation versus 21% (95% CI 19%–24%) for continuation. The absolute 2‐year risk increase was thus 21% (95% CI 18%–26%).

### 3.3 Evaluating what patient factors modify the influence of discontinuation on seizure risk (“Individual Interactions Model”)

Age at start of follow‐up (*p* = 0.05) and seizures impairing awareness (*p* = 0.02) significantly increased the odds ratio for the effect of discontinuation on seizures. Table 1 contains all interaction *p*‐values. The odds ratio for a seizure in any given month comparing discontinuation versus continuation was 0.9 (95% CI 0.8–1.0) at age 30 years. This odds ratio increased by a factor of 1.2 (95% CI 1.0–1.4) for every decade, suggesting that discontinuation had a relatively larger effect for older patients. For example, this would imply a predicted odds ratio due to discontinuation of 0.9 × 1.2^2^ = 1.3 at age of 50 years and an odds ratio of 0.9 × 1.2^4^ = 1.8 at age of 70. The odds ratio for seizures in any given month due to discontinuation was 0.7 (95% CI 0.5–1.0) in the absence of seizures impairing awareness, versus 1.2 in the presence of seizures impairing awareness.

However, after setting the false discovery rate to 5%, no corrected, adjusted *p*‐values remained significant. Also, there was little difference in discrimination (area under the curve: 0.72 for the base model and 0.73 for individual interactions model) or calibration (Figure S1) depending on whether interactions were included. Thus, we eliminated all individual interactions from the subsequent risk interaction model, below.

### 3.4 Evaluating the distribution of seizure risk increase due to discontinuation across patients (“Risk Interaction Model”)

The median (IQR) predicted risk of any seizure by 2 years was as follows (Figure S2): 39% (28%–50%) for discontinuation versus 19% (13%–26%) for continuation. Discontinuation increased the absolute 2‐year seizure risk by a median of 19% (IQR 14%–24%).

**FIGURE 2:**
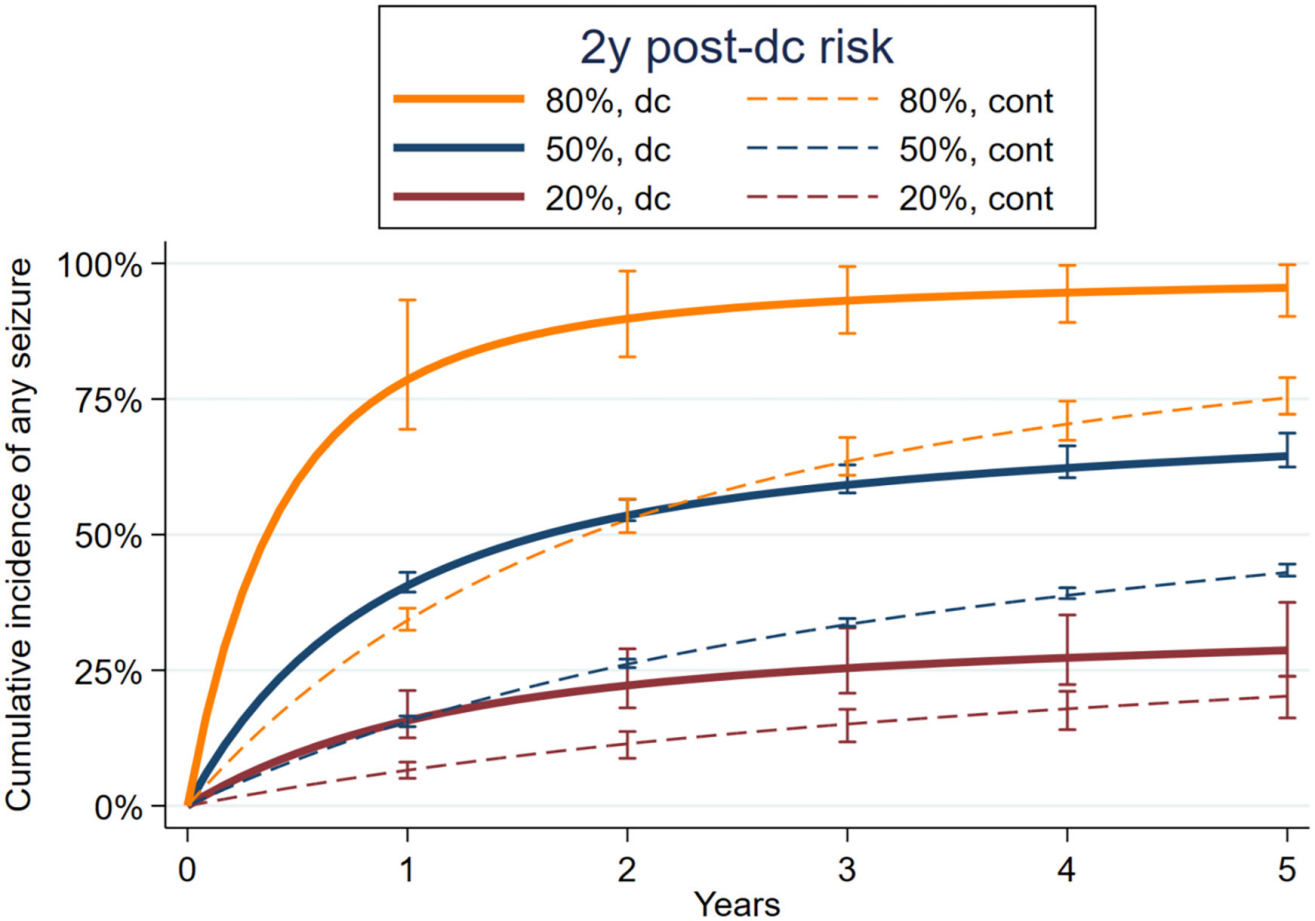
Adjusted cumulative seizure incidence over time for discontinuation versus continuation. For patients at several representative hypothetical 2‐year post‐discontinuation seizure risks per the base model, curves were drawn from the risk interaction model. Error bars represent bootstrapped 95% confidence intervals. “cont”, continuation; “dc”, post‐discontinuation.

We calculated survival curves for several hypothetical, representative seizure risks (Figure 2). For hypothetical patients with base model predicted 2‐year discontinuation seizure risks of 20%, 50%, and 80%, the risk interaction model found discontinuation 2‐year versus continuation risks of 22% versus 11% (risk difference = 11%), 54% versus 26% (risk difference = 28%), and 89% versus 53% (risk difference = 36%), respectively.

**FIGURE 3:**
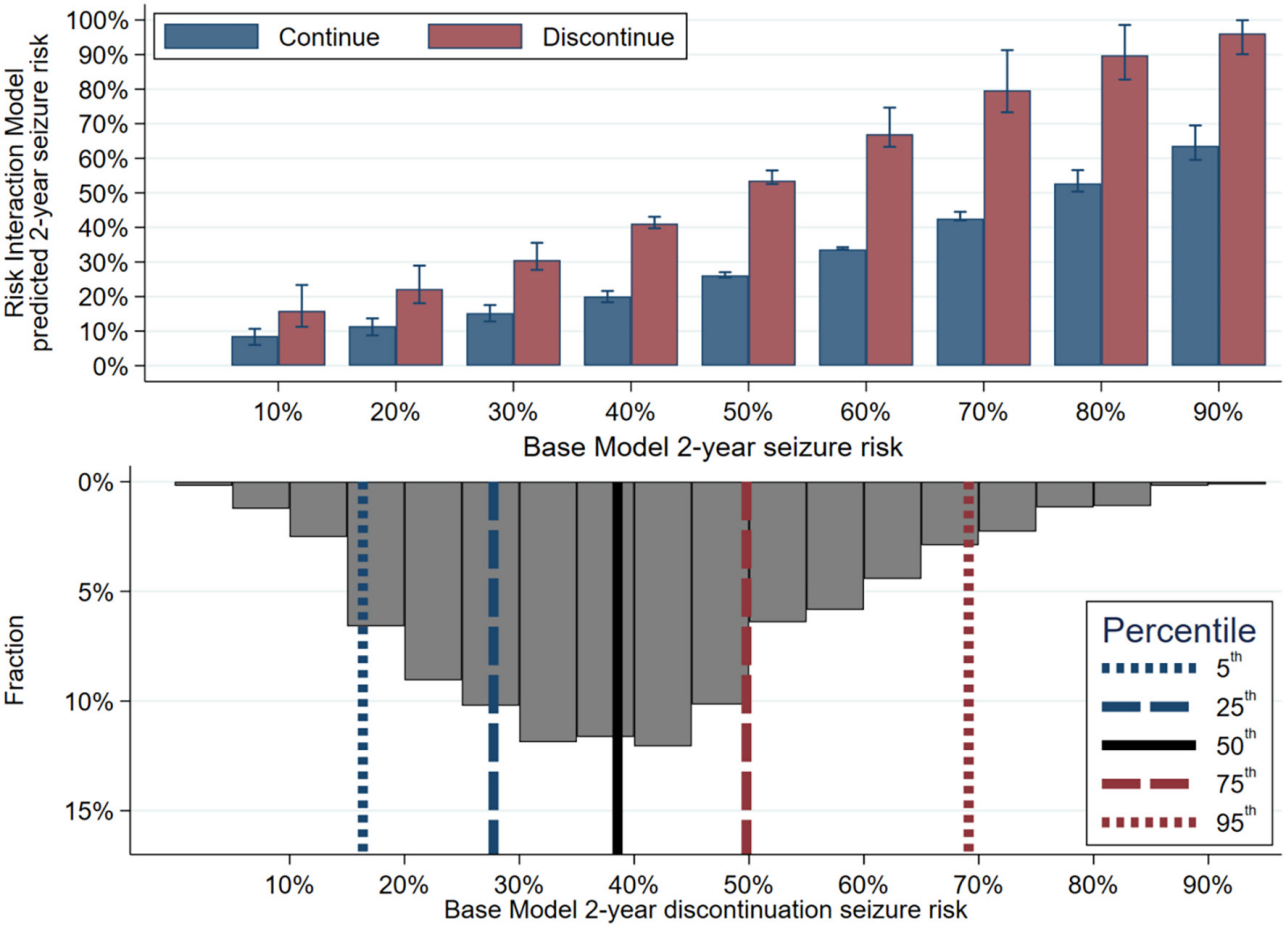
Absolute 2‐year seizure probabilities for discontinuation versus continuation. Top: Predictions from the risk interaction model for different basel model risks. We superimposed a histogram immediately below to show the distribution of where most of our sample falls on the top figure's *X*‐axis. Error bars represent bootstrapped 95% confidence intervals.

We then calculated 2‐year seizure risks, across the entire risk spectrum. Figure 3 displays absolute risks across the risk spectrum (top) plus a superimposed histogram describing the risk distribution of our sample (bottom). Table 2A further describes these results, including that the absolute seizure risk increase ranged from 7% to 37%, and the odds ratio increased with increasing risk levels (*p* < 0.01).

**TABLE 2:** Effects of discontinuation on 2‐year seizure risk.

Base model hypothetical 2‐year discontinuation risk (%)	Risk interaction model 2‐year discontinuation risk (%)	Risk interaction model 2‐year continuation risk (%)	Absolute risk increase (%)	Odds ratio	Number needed to harm
(A) All three studies					
10	16 (11–23)	9 (6–11)	7 (3–14)	2.0 (1.3–3.6)	13.7 (6.1–37.7)
20	22 (18–29)	11 (9–14)	11 (6–17)	2.2 (1.6–3.5)	9.3 (5.8–15.4)
30	31 (28–36)	15 (13–18)	15 (12–21)	2.5 (1.9–3.5)	6.5 (4.7–8.4)
40	41 (40–43)	20 (18–22)	21 (18–26)	2.8 (2.3–3.6)	4.7 (3.9–5.4)
50	54 (53–56)	26 (25–27)	27 (23–33)	3.3 (2.6–4.3)	3.6 (3.0–4.3)
60	67 (63–75)	34 (34–34)	33 (26–44)	4.0 (2.9–6.7)	3.0 (2.3–3.9)
70	80 (73–91)	43 (42–45)	37 (26–50)	5.3 (3.2–13.1)	2.7 (2.0–3.9)
80	90 (83–99)	53 (50–57)	37 (21–48)	7.9 (3.7–40.7)	2.7 (2.1–4.7)
90	96 (90–100)	64 (60–69)	33 (13–43)	14.2 (4.5–279)	3.1 (2.4–8.0)
(B) Only the two RCTs					
10	15 (9–21)	8 (4–11)	7 (2–16)	2.0 (1.2–4.3)	14.1 (6.2–56.5)
20	22 (16–29)	11 (7–14)	10 (5–18)	2.2 (1.5–3.8)	9.6 (5.4–18.7)
30	30 (27–37)	16 (13–19)	15 (11–22)	2.4 (1.9–3.5)	6.7 (4.6–8.9)
40	42 (41–44)	21 (19–24)	20 (17–25)	2.6 (2.1–3.3)	4.9 (4.0–5.7)
50	55 (55–57)	29 (27–33)	26 (22–35)	3.0 (2.4–4.7)	3.8 (2.8–4.6)
60	69 (67–75)	38 (35–47)	31 (22–48)	3.6 (2.5–8.1)	3.2 (2.1–4.5)
70	82 (80–92)	50 (44–62)	33 (17–53)	4.8 (2.7–17.2)	3.0 (1.9–6.0)
80	92 (88–99)	62 (53–79)	30 (6–49)	7.1 (2.9–56.8)	3.3 (2.0–14.5)
90	97 (95–100)	74 (63–92)	23 (−4 to 40)	13.3 (3.6–356)	4.3 (2.5–334)

*Note:* We display absolute risk increases in discontinuation versus continuation, odds ratios, and number needed to harm (to produce at least one seizure relapse within 2 years due to discontinuation in one additional patient) for patients at various levels of risk. The left column is the base model‘s hypothetical discontinuation risk. We entered that quantity into the risk interaction model, in which we allowed the relative effect of discontinuation to vary across patients with different base model risks, hence the base model hypothetical discontinuation risk is not identical to the risk interaction model‘s predicted discontinuation risk.

We conducted several sensitivity analyses. Base model 2‐year discontinuation predictions correlated moderately with Lamberink model predictions (Spearman's ρ = 0.61; 95% CI 0.55–0.67; Figure S4). Two‐year discontinuation predicted probabilities from the Lamberink model had a median of 66% (IQR 49%–81%; Figure S2). Survival curves using Lamberink model predictions instead of base model predictions (Figure S3, left; *n* = 464) or when using RCT data only (Figure S3, right; *n* = 1161) were similar to the main analyses. Sensitivity analyses restricted to only RCT data further demonstrated little qualitative change compared with our main results (Table 2B and Figure S5).

## 4 DISCUSSION

We quantified variation in seizure risk increase due to ASM discontinuation, after a period of seizure freedom. The effect of discontinuation did not strongly depend on any single risk factor. Instead, as expected, a patient's overall risk, after accounting for all risk factors, did strongly determine the absolute risk increase associated with ASM discontinuation. While it can be intuited qualitatively that patients with higher absolute risk likely experience greater absolute risk reduction from treatment, our study's key contribution was to quantify the distribution of absolute risk increases across the risk spectrum. This sort of quantitative understanding of an individual patients treatment effect is needed when deciding whether the absolute benefit outweighs the downsides of treatment. Another insight from this study is that not only did absolute discontinuation seizure risk increase for patients at higher risk but also relative seizure risks increased with increasing risk. This suggests that higher‐risk patients may derive greater both absolute and relative benefit from ASMs and identifies a low‐risk population for whom discontinuation may be particularly considered given treatment effects even smaller than might have been predicted by group average treatment effects alone.

Identifying patients with both low absolute and low relative treatment effects would enable clinicians to target patients for safest discontinuation. Our work represents one step along this larger goal—to develop an enhanced individualized seizure risk calculator including both discontinuation and continuation estimates. We will use this study's findings (ie, absence of strong interactions between discontinuation and individual factors, but interaction with post‐discontinuation risk) to inform our future modeling strategies. We are planning future studies to hopefully improve upon post‐discontinuation seizure prediction, which would be useful given the existing model^13^, ^14^ demonstrated moderate discrimination (area under the curve 0.65) during internal‐external validation, followed by moderate‐to‐poor discrimination (areas under the curve ranging from 0.51 to 0.71) and some overprediction during external validation^31^, ^32^, ^33^ in addition to overprediction in our datasets. Then, we will be able to apply the discontinuation effects observed in the current study to derive a two‐armed calculator (not just one armed, as is the current calculator^14^) making the crucial comparison for a particular individual regarding what their seizure risk might be if they continued versus discontinued ASMs.

Even if one could estimate counterfactual risks perfectly, however, we acknowledge that determining what constitutes an acceptably “low” risk increase remains a challenge. Risk is never zero, regardless of whether the patient is treated, it is always possible that a patient may have difficulty regaining seizure control after a relapse, and all patients and clinicians will have a different level of risk tolerance. We recently surveyed American Academy of Neurology Members who indicated that they would tolerate a median (IQR) post‐discontinuation risk of 15% (IQR 9%–29%) for convulsive seizures and 24% (IQR 11%–35%) for nonconvulsive seizures in adults.^34^ In our sample, only 18% had a post‐discontinuation risk per the base model below 24%. Still, epilepsy is diagnosed when 10‐year seizure risk rises to at least 60%. Although available datasets generally do not contain such long‐term follow‐up, as a rough estimation, given survival curves substantially level off after the first several years, we note that 76% of our population had a 2‐year post‐discontinuation predicted seizure rate of less than 50%, thus there may be a sizeable population who could be discontinuation candidates.

Regardless, we have learned from this study that, given 2‐year absolute treatment effects ranged from 7% to 37%, whenever the downsides of ASMs are felt to not be worth a substantial seizure reduction (absolute risk reduction over 37%, or number needed to harm less than 3), the patient should probably discontinue. However, if the downsides of ASMs are felt to be minimal and the patient would accept remaining on ASMs even if they provided only minimal seizure reduction (absolute risk reduction less than 7%, or number needed to harm at least 14), it would seem justified to continue in most cases. Many patients' values and preferences will fall between these two extremes, in which case having an accurate individualized seizure risk prediction for such patients could directly inform decision‐making. Of course, the above statistics are only estimates and it is quite possible that patients may fall outside of these bounds depending on an expert clinician's knowledge of the patient based on factors that were not available as variables in our datasets. However, our work provides the best data‐driven estimates to date of variation in discontinuation effects. In our future work, we will use what we have learned in the current study to develop our results into an individualized risk prediction calculator to assist with decision‐making for patients in whom intermediate seizure probabilities would matter, and we hope to conduct future work improving our knowledge of how low seizure risk may need to be to confer benefit to discontinuation across different patient phenotypes.

Increasing age predicted an increasing relative effect of discontinuation. We had hypothesized that increasing age might predict decreasing effects from discontinuation, given some literature suggesting that older patients might be able to be treated with lower doses, thus “easier to treat.”^35^, ^36^, ^37^, ^38^ A relationship between older age and relative effects of seizure discontinuation would be critical information, if true, given older adults have the highest incidence of epilepsy compared with any other time in life.^39^ However, interpreting this significant result requires caution. This was one of many exploratory analyses and was no longer significant after accounting for multiple comparisons. Also, we entered age as a linear interaction term—the true biological effects of age may be more complex. Future work should seek to disentangle age versus factors associated with age, such as differential baseline adherence. Additionally, if older patients in our sample were treated with similar doses as younger patients, they could have seen effectively greater serum concentrations due to impaired hepatorenal metabolism, which would be yet one more reason explaining the apparent increased efficacy of ASMs. Our work has begun exploring these important yet complex possible interactions and will require future validation.

### 4.1 Our work has limitations

First, intention to discontinue (eg, being randomized, or deciding at a clinic visit) is not identical to actually discontinuing and thus our findings could understate the influence of discontinuation. However, we previously demonstrated that the effect of nonadherence to randomized arm in MRC was trivial,^16^ and adherence to randomized arms was nearly perfect in Lossius data.^11^

Second, each interaction must be interpreted in the setting of included confounders and modeling assumptions, it is not possible to test for all possible higher‐order effects or interactions, and no dataset in existence has measured all possibly relevant variables. For example, datasets did not contain lifestyle factors (eg, sleep, substance use, and alcohol) or baseline ASM adherence. We see this as an opportunity for future prospective data collection and emphasize that we included among the strongest datasets currently in existence for this question. Also, heterogeneity is a strength in risk prediction work to the extent that it has been captured—we measured many important dimensions such as demographics, semiology, focality, prior intracranial surgery, EEG results, ASM characteristics, epilepsy and seizure‐free durations, and more. Although we acknowledge that future work more completely specifying a patient's particular self‐limited epilepsy syndrome would be helpful, as this was not specified in the randomized data.

Third, in real clinical practice, discontinuation versus continuation are not the only options. Clinicians can also switch ASMs or partially reduce treatment. Our prior work produced the first‐ever dose‐response curves regarding ASM discontinuation to explore how seizure risk changes across partial dose reductions.^16^

Fourth, each dataset has limitations. For example, while clinical trials are not confounded, patients enrolled in clinical trials may not exactly represent its intended source population due to the nature of recruitment and structured observation, and the Lossius et al trial enrolled an unusually low‐risk population. However, the MRC study was conducted across 40 sites in the United Kingdom, supporting its generalizability to its enrolled population, and the Lossius study provides the highest quality evidence to date given it was double blinded. In the observational chart review, unmeasured factors related to seizure risk may have influenced the decision to withdraw. Still, our observational chart review provides real‐world multicenter data, we specifically sought to reduce selection bias by studying all patients (not just discontinuers) including a variable for the time of discontinuation if any, and ultimately results were unchanged when excluding this observational cohort. However, we acknowledge these data were collected from academic centers that may not reflect patients and practices in community centers.

## 5 CONCLUSIONS

Many patients with epilepsy attaining a seizure‐free period may have acceptably low risk to eventually attempt ASM discontinuation, and our study highlights marked variation in discontinuation effects as a step toward identifying those patients. No individual variable robustly predicted relative differences in the influence of discontinuation on seizure risk. Increasing age and seizures impairing awareness predicted a greater relative influence of discontinuation on seizure relapses in uncorrected analysis, but not after correcting for multiple comparisons. Our results will be useful to clinicians by demonstrating the range of effects that might be expected from discontinuation in the population. Our future work will seek to contextualize optimal seizure risk thresholds below which discontinuation may be considered and develop a two‐armed point‐of‐care seizure risk calculator applying what we have learned from this study.

## AUTHOR CONTRIBUTIONS

S. W. T. conceived of and designed the study, collected data, executed the statistical analysis, and wrote the manuscript. G. S. designed the study and collected and interpreted the data. A. K., J. S., and M. V. S. collected data. All authors edited the manuscript. J. F. B., R. D. T., and K. P. J. B. provided study supervision.

## FUNDING INFORMATION

These funding sources had no role in the planning or execution of this project. Dr Terman was recently supported by the Susan S Spencer Clinical Research Training Scholarship and the Michigan Institute for Clinical and Health Research J Award UL1TR002240. Dr Terman is now supported by an American Epilepsy Society Research and Training Fellowship for Clinicians. Dr Terman was a member of the Junior Investigator Intensive Program of the US Deprescribing Research Network, which is funded by the National Institute on Aging (R24AG064025). Dr Slinger is supported by the friends UMC Utrecht/MING Fund. Dr Springer is supported by the National Institutes of Health K01 NS117555. Dr Ziobro is supported by the PCHD19 Alliance/American Epilepsy Society Research Training Fellowship for Clinicians and the National Institutes of Health K08 NS124937. Dr Otte is supported by the Dutch Epilepsy Fund and the friends UMC Utrecht/MING Fund. Dr Thijs reports lecture and consultancy fees from Medtronic, UCB, Theravarance, Zogenix, Novartis, and Arvelle, and grants from EpilepsieNL, Medtronic, Michael J Fox Foundation, NewLife Wearables and Health‐Holland, Top Sector Life Sciences & Health Netherlands Organization for Health Research and Development (ZonMW) [Brain@home, Project number: 114025101], and the Christelijke Vereniging voor de Verpleging van Lijders aan Epilepsie. Dr Skvarce, Dr Koek, Dr Burke, Dr Lossius, Prof Marson, Dr Bonnett and Dr Braun reports no relevant funding.

## CONFLICT OF INTEREST STATEMENT

None of the authors has any conflict of interest to disclose. We confirm that we have read the Journal's position on issues involved in ethical publication and affirm that this report is consistent with those guidelines.

## Supporting information


Appendix S1.

## Data Availability

Statistical code for this project may be obtained from the authors. Data can only be shared with agreement from primary investigators and data use agreements where applicable.

## References

[1] Epilepsy: a public health imperative [Internet]. Geneva: World Health Organization; 2019 [cited 2023]. Available from: https://www.who.int/mental_health/neurology/epilepsy/report_2019/en/

[2] Chen Z , Brodie MJ , Liew D , Kwan P . Treatment outcomes in patients with newly diagnosed epilepsy treated with established and new antiepileptic drugs a 30‐year longitudinal cohort study. JAMA Neurol. 2018;75(3):279–286. 29279892 10.1001/jamaneurol.2017.3949 PMC5885858

[3] Hamilton KJ , Chen Z , Tomlin A , Kwan P . Mortality and morbidity of patients with treated and untreated epilepsy in New Zealand. Epilepsia. 2020;61(3):519–527. 31981218 10.1111/epi.16435

[4] Perucca P , Gilliam FG . Adverse effects of antiepileptic drugs. Lancet. 2012;11(September):792–802. 10.1016/S1474-4422(12)70153-9 22832500

[5] Jacoby A , Snape D , Baker GA . Determinants of quality of life in people with epilepsy. Neurol Clin. 2009;27(4):843–863. 19853212 10.1016/j.ncl.2009.06.003

[6] Lamberink HJ , Otte WM , Geleijns K , Braun KPJ . Antiepileptic drug withdrawal in medically and surgically treated patients: a meta‐analysis of seizure recurrence and systematic review of its predictors. Epileptic Disord. 2015;17(3):211–228. 26292909 10.1684/epd.2015.0764

[7] Beghi E , Giussani G , Grosso S , Iudice A , La A , Pisani F , et al. Withdrawal of antiepileptic drugs: guidelines of the Italian league against epilepsy. Epilepsia. 2013;54(Suppl 7):2–12. 10.1111/epi.12305 24099051

[8] American Academy of Neurology . Practice Parameter: a guideline for discontinuing antiepileptic drugs in seizure‐free patients—Summary Statement. Report of the Quality Standards Subcommittee of the American Academy of Neurology. Neurology. 1996;47(2):600–602. 8757050 10.1212/wnl.47.2.600

[9] Gloss D , Pargeon K , Pack A , Varma J , French JA , Tolchin B , et al. Antiseizure medication withdrawal in seizure‐free patients: practice advisory update summary. Neurology. 2021;97(23):1072–1081. 34873018 10.1212/WNL.0000000000012944

[10] Medical Research Council . Randomised study of antiepileptic drug withdrawal in patients in remission. Lancet. 1991;337(8751):1175–1180. 1673736

[11] Lossius MI , Hessen E , Mowinckel P , Stavem K , Erikssen J , Gulbrandsen P , et al. Consequences of antiepileptic drug withdrawal: a randomized, double‐blind study (Akershus study). Epilepsia. 2008;49(3):455–463. 17888074 10.1111/j.1528-1167.2007.01323.x

[12] Wang J , Huang P , Song Z . Comparison of the relapse rates in seizure‐free patients in whom antiepileptic therapy was discontinued and those in whom the therapy was continued: a meta‐analysis. Epilepsy Behav. 2019;101(Pt A):106577. 31706169 10.1016/j.yebeh.2019.106577

[13] Lamberink HJ , Otte WM , Geerts AT , Pavlovic M , Ramos‐Lizana J , Marson AG , et al. Individualised prediction model of seizure recurrence and long‐term outcomes after withdrawal of antiepileptic drugs in seizure‐free patients: a systematic review and individual participant data meta‐analysis. Lancet Neurol. 2017;16(7):523–531. 28483337 10.1016/S1474-4422(17)30114-X

[14] AED Withdrawal Risk Calculator [Internet]. [cited 2023]. Available from: http://epilepsypredictiontools.info/aedwithdrawal

[15] Chadwick D . Prognostic index for recurrence of seizures after remission of epilepsy. BMJ. 1993;306(6889):1374–1378. 8518603 10.1136/bmj.306.6889.1374 PMC1677783

[16] Terman SW , Wang C , Wang L , Braun KPJ , Otte WM , Slinger G , et al. Reappraisal of the Medical Research Council antiepileptic drug withdrawal study: contamination‐adjusted and dose–response re‐analysis. Epilepsia. 2022;63(7):1724–1735. 35490396 10.1111/epi.17273 PMC9283317

[17] Terman SW , Slinger G , Koek A , Skvarce J , Springer MV , Ziobro JM , et al. Frequency of and factors associated with antiseizure medication discontinuation discussions and decisions in patients with epilepsy: a multicenter retrospective chart review. Epilepsia Open. 2023;8(2):371–385. 36693718 10.1002/epi4.12695 PMC10235583

[18] Hernán MA . The hazards of hazard ratios. Epidemiology. 2010;21(1):13–15. 20010207 10.1097/EDE.0b013e3181c1ea43 PMC3653612

[19] Hernan MA , Robins JM . Chapter 17: causal survival analysis. 17.2: from hazards to risks. What if. Boca Raton: CRC Press; 2020. p. 211–214. https://www.hsph.harvard.edu/miguel‐hernan/causal‐inference‐book/

[20] White IR , Royston P , Wood AM . Multiple imputation using chained equations: issues and guidance for practice. Stat Med. 2011;30(4):377–399. 21225900 10.1002/sim.4067

[21] Little RJ , Rubin DB . Statistical analysis with missing data. 3rd ed. Hoboken, NJ: Wiley; 2019.

[22] Rubin DB , Schenker N , Rubin DB , Schenker N . Ignorable nonresponse multiple imputation for interval estimation from simple random samples with ignorable nonresponse. J Am Stat Assoc. 1986;81(394):366–374.

[23] Von Hippel PT . How to impute interactions, squares, and other transformed variables. Sociol Methodol. 2009;39(1):265–291.

[24] Benjamini Y , Hochberg Y . Controlling the false discovery rate: a practical and powerful approach to multiple testing. J R Stat Soc. 1995;57(1):289–300.

[25] Lagakos SW . The challenge of subgroup analyses—reporting without distorting. NEJM. 2006;354(16):1667–1670. 16625007 10.1056/NEJMp068070

[26] Kent DM , Steyerberg E , van Klaveren D . Personalized evidence based medicine: predictive approaches to heterogeneous treatment effects. BMJ. 2018;363:k4245. 30530757 10.1136/bmj.k4245 PMC6889830

[27] Kent DM , Hayward RA . Limitations of applying summary results of clinical trials to individual patients: the need for risk Stratificiation. JAMA. 2007;298(10):1209–1212. 17848656 10.1001/jama.298.10.1209

[28] Goligher EC , Lawler PR , Jensen TP , Talisa V , Berry LR , Lorenzi E , et al. Heterogeneous treatment effects of therapeutic‐dose heparin in patients hospitalized for COVID‐19. JAMA. 2023;329(13):1066–1077. 36942550 10.1001/jama.2023.3651 PMC10031504

[29] Hayward RA , Kent DM , Vijan S , Hofer TP . Multivariable risk prediction can greatly enhance the statistical power of clinical trial subgroup analysis. BMC Med Res Methodol. 2006;6:18. 16613605 10.1186/1471-2288-6-18 PMC1523355

[30] van Klaveren D , Balan TA , Steyerberg EW , Kent DM . Models with interactions overestimated heterogeneity of treatment effects and were prone to treatment mistargeting. J Clin Epidemiol. 2019;114:72–83. 31195109 10.1016/j.jclinepi.2019.05.029 PMC7497896

[31] Lin J , Ding S , Li X , Hua Y , Wang X , He R , et al. External validation and comparison of two prediction models for seizure recurrence after the withdrawal of antiepileptic drugs in adult patients. Epilepsia. 2020;61(1):115–124. 31792957 10.1111/epi.16402

[32] Contento M , Bertaccini B , Biggi M , Magliani M , Failli Y , Rosati E , et al. Prediction of seizure recurrence risk following discontinuation of antiepileptic drugs. Epilepsia. 2021;62(9):2159–2170. 34250596 10.1111/epi.16993 PMC8457060

[33] Chu S‐S , Tan G , Wang X‐P , Liu L . Validation of the predictive model for seizure recurrence after withdrawal of antiepileptic drugs. Epilepsy Behav. 2021;114:106987. 10.1016/j.yebeh.2020.106987 32444329

[34] Terman SW , Slinger G , Rheaume CE , Haque AS , Smith SN , van Griethuysen R , et al. Antiseizure medication withdrawal practice patterns: a survey among members of the American Academy of Neurology and EpiCARE. Neurol Clin Pract. 2023;13(1):e200109. 37063781 10.1212/CPJ.0000000000200109 PMC10101711

[35] Acharya JN , Acharya VJ . Epilepsy in the elderly: special considerations and challenges. Ann Indian Acad Neurol. 2014;17(Supplement 1):S19–S26. 10.4103/0972-2327.128645 PMC4001216 24791083

[36] Stefan H . Epilepsy in the elderly: facts and challenges. Acta Neurol Scand. 2011;124(4):223–237. 21143593 10.1111/j.1600-0404.2010.01464.x

[37] Cockerell OC , Johnson AL , Sander JWAS , Shorvon SD . Prognosis of epilepsy: a review and further analysis of the first nine years of the British National General Practice Study of epilepsy, a prospective population‐based study. Epilepsia. 1997;38(1):31–46. 9024182 10.1111/j.1528-1157.1997.tb01075.x

[38] Sarkis RA , Beers L , Farah E , Al‐Akaidi M , Zhang Y , Locascio JJ , et al. The neurophysiology and seizure outcomes of late onset unexplained epilepsy. Clin Neurophysiol. 2020;131(11):2667–2672. 10.1016/j.clinph.2020.08.014 32957039 PMC7644268

[39] Hauser WA , Annegers JF , Kurland LT . Incidence of epilepsy and unprovoked seizures in Rochester, Minnesota: 1935–1984. Epilepsia. 1993;34(3):453–468. 8504780 10.1111/j.1528-1157.1993.tb02586.x

[40] Motor seizure [Internet]. International League Against Epilepsy. 2022 [cited 2023]. Available from: https://www.epilepsydiagnosis.org/seizure/motor‐overview.html

