# Supplementary material for: Variation in seizure risk increases from antiseizure medication withdrawal among patients with well‐controlled epilepsy: A pooled analysis
Source: Epilepsia Open. 2023 Dec 16;9(1):333–44. doi: 10.1002/epi4.12880 (PMC10839298; doi:10.1002/epi4.12880)
Supplement: Supplementary file 1 — Appendix S1. [file EPI4-9-333-s001.zip › table S1-3.docx]

**Supplemental Table 1**: Variables available in each study. Numbers in each cell represent the number of patients in each dataset with an available value for each listed variable.

| **Variable** | **All**  **(n=1,626)** | **MRC (n=1,013)** | **Lossius**  **(n=149)** | **Retrospective (n=464)** | **Lamberink calculator variables** |
| --- | --- | --- | --- | --- | --- |
| Age at start of follow-up | 1,626 | 1,013 | 149 | 464 |  |
| ASM names, number, old versus new generation | 1,626 | 1,013 | 149 | 464 |  |
| Structural etiology ** | 1,626 | 1,013 | 149 | 464 |  |
| Female | 1,626 | 1,013 | 149 | 464 |  |
| Developmental delay | 1,625 | 1,013 | 149 | 463 | **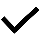** |
| Epileptiform EEG* | 1,625 | 1,013 | 148 | 464 | **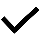** |
| Seizures impairing awareness | 1,625 | 1,013 | 149 | 463 |  |
| Motor seizures | 1,625 | 1,013 | 149 | 463 |  |
| Years seizure-free category | 1,625 | 1,012*** | 149 | 464 |  |
| Focal epilepsy | 1,617 | 1,013 | 149 | 455 |  |
| Age at epilepsy diagnosis category | 1,614 | 1,013 | 149 | 452 |  |
| Prior discontinuation attempt | 1,519 | 1,013 | 49 | 457 |  |
| Brain surgery | 1,477 | 1,013 | 0 | 464 |  |
| Years seizure-free | 1,477 | 1,013 | 0 | 464 | **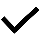** |
| Family history of seizures | 1,475 | 1,011 | 0 | 464 |  |
| Febrile seizures | 1,473 | 1,009 | 0 | 464 | **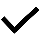** |
| Age at epilepsy diagnosis | 1,465 | 1,013 | 0 | 452 | **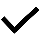** |
| Years seizures before remission | 1,465 | 1,013 | 0 | 452 | **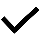** |
| Abnormal neurological exam | 1,162 | 1,013 | 149 | 0 |  |
| Self-limited syndrome | 458 | 0 | 0 | 458 | **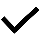** |
| >9 lifetime seizures | 456 | 0 | 0 | 456 | **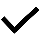** |
| Prior status epilepticus | 451 | 0 | 0 | 451 |  |

*EEG was considered all present, given lack of performing an EEG remains informative per the Lamberink calculator thus missing was coded as an informative value.

**We entered structural etiology as the ‘etiology’ variable, given small sample sizes for any single specific structural etiologies and each non-structural etiology other than ‘unknown.’

***One patient in MRC was recorded as being 13 months seizure-free at study onset, despite the requirement to be 24 months seizure-free for all patients. Thus, given this may have represented a data entry error during the original study several decades ago with no ability to correct this value, we recorded this patient as missing a years seizure-free category given doubt about the accuracy of that value.

**Supplemental Table 2:** Patient population at start of follow-up, by dataset. Blanks exist given not all datasets contained all variables. Several variables include both categorical and continuous descriptions because Lossius et al. measured several variables only as categories, whereas other datasets had continuous measurements available.

|  | | **Median (IQR) or No. (%)** | | |
| --- | --- | --- | --- | --- |
|  | | **MRC**  **N=1,013** | **Lossius**  **N=149** | **Retrospective**  **N=464** |
| Age at epilepsy diagnosis |  | 14 (7-23) | - | 14 (7-23) |
| Age at epilepsy diagnosis | <11 | 382 (38%) | 14 (9%) | 192 (42%) |
| category | 11-17 | 250 (25%) | 43 (29%) | 102 (23%) |
|  | 18+ | 381 (38%) | 92 (62%) | 158 (35%) |
| Age at start of follow-up |  | 27 (17-41) | 38 (29-47) | 33 (18-53) |
| Years seizures before remission | | 5 (1-12) | **-** | 7 (2-20) |
| Female |  | 516 (51%) | 79 (53%) | 221 (48%) |
| Years seizure-free |  | 3 (2-6) | - | 2 (1-5) |
| Years seizure-free category | <2 | 0 (0%) | 0 (0%) | 206 (44%) |
|  | 2 to <3 | 427 (42%) | 9 (6%) | 87 (19%) |
|  | 3 to <5 | 263 (26%) | 40 (27%) | 62 (13%) |
|  | 5+ | 322 (32%) | 100 (67%) | 109 (23%) |
| Focal epilepsy |  | 320 (32%) | 114 (77%) | 359 (79%) |
| Seizures impairing awareness |  | 327 (32%) | 78 (52%) | 452 (98%) |
| Epileptiform EEG |  | 167 (16%) | 70 (47%) | 217 (47%) |
| Number | 1 | 842 (83%) | 149 (100%) | 262 (56%) |
| of ASMs | 2 | 161 (16%) | 0 (0%) | 146 (31%) |
|  | 3+ | 10 (1%) | 0 (0%) | 56 (12%) |
| Motor seizures | | 876 (86%) | 126 (85%) | 418 (90%) |
| Older generation ASM | | 1,008 (100%) | 143 (96%) | 260 (56%) |
| Newer generation ASM | | 12 (1%) | 6 (4%) | 312 (67%) |
| Structural etiology** | | 138 (14%) | 38 (26%) | 122 (26%) |
| Prior discontinuation attempt | | 118 (12%) | 0 (0%)* | 144 (32%) |
| Brain surgery | | 17 (2%) | **-** | 32 (7%) |
| Family history of seizures | | 152 (15%) | **-** | 105 (23%) |
| Febrile seizures | | 100 (10%) | **-** | 42 (9%) |
| >9 lifetime | | **-** | **-** | 366 (80%) |
| Self-limited syndrome | | **-** | **-** | 17 (4%) |
| Developmental delay | | 153 (15%) | 0 (0%) | 82 (18%) |
| Prior status epilepticus | | - | **-** | 105 (23%) |

*Lossius et al excluded patients if fewer than five years seizure-free with any prior discontinuation attempt with a subsequent relapse. Therefore, all included patients who were fewer than five years seizure-free (49) had no prior discontinuation attempt. However, the study did not collect prior discontinuation attempt as a variable for included patients and thus this variable was missing for the 100 included patients who were seizure-free over five years. This is reflected in Supplemental Table 2 as well.

**Supplemental Table 3**: Base Model coefficients. This was from a discrete time logistic regression in which the outcome was whether a seizure occurred that month, each row represented one person-month, and people had as many rows as they had months of follow-up until their first seizure, lost to follow-up, or else the end of the study period (whichever came first). Coefficients less than 0 signify a negative predictor, whereas coefficients greater than 0 signify a positive predictor. All coefficients listed in this Table are adjusted for all other coefficients listed in this Table. To obtain odds ratios, these coefficients would need to be exponentiated. Bold font signifies p<0.05.

| **Variable** | **Coefficient** | **95% CI** |
| --- | --- | --- |
| Time | -0.017 | -0.056 to 0.021 |
| Time^2^ | 0.000 | -0.002 to 0.001 |
| Time^3^ | 0.000 | 0.000 to 0.000 |
| **Discontinue** | **1.349** | **0.982 to 1.715** |
| Discontinue*Time | -0.053 | -0.109 to 0.003 |
| Discontinue*Time^2^ | 0.000 | -0.002 to 0.002 |
| Discontinue*Time^3^ | 0.000 | 0.000 to 0.000 |
| **Focal epilepsy** | **-0.292** | **-0.491 to -0.093** |
| Motor seizures | 0.241 | -0.066 to 0.549 |
| Developmental delay | 0.131 | -0.154 to 0.417 |
| Febrile seizures | 0.227 | -0.047 to 0.502 |
| Prior discontinuation attempt | -0.028 | -0.271 to 0.216 |
| Family history of seizures | 0.146 | -0.068 to 0.360 |
| >9 lifetime seizures | -0.089 | -0.655 to 0.477 |
| Self-limited syndrome | -0.722 | -1.773 to 0.328 |
| Prior status epilepticus | 0.272 | -0.106 to 0.649 |
| Age at epilepsy diagnosis category |  |  |
| <11 | Reference |  |
| **11-17** | **0.330** | **0.098 to 0.562** |
| **18+** | **0.313** | **0.021 to 0.605** |
| **Years seizure-free** | **-0.089** | **-0.118 to -0.06** |
| **Log(years seizures)** | **0.117** | **0.010 to 0.224** |
| Female | 0.132 | -0.035 to 0.299 |
| Number of ASMs |  |  |
| 1 | Reference |  |
| **2** | 0.499 | 0.276 to 0.723 |
| 3+ | 0.380 | -0.057 to 0.816 |
| Older generation ASM | -0.249 | -0.57 to 0.072 |
| Structural etiology | 0.074 | -0.16 to 0.308 |
| Epileptiform EEG |  |  |
| No | Reference |  |
| Yes | 0.183 | -0.017 to 0.383 |
| Unknown | Reference |  |
| Age at start of follow-up, per decade | -0.041 | -0.126 to 0.044 |
| Study |  |  |
| MRC | Reference |  |
| **Lossius** | **-0.530** | **-1.047 to -0.013** |
| Chart review | 0.199 | -0.127 to 0.526 |
| Seizures impairing awareness | -0.025 | -0.237 to 0.187 |
| Abnormal neurological exam | -0.142 | -0.518 to 0.234 |
| **Constant** | **-4.550** | **-5.243 to -3.857** |
