# Supplementary figures and images for: Variation in seizure risk increases from antiseizure medication withdrawal among patients with well‐controlled epilepsy: A pooled analysis
Source: Epilepsia Open. 2023 Dec 16;9(1):333–44. doi: 10.1002/epi4.12880 (PMC10839298; doi:10.1002/epi4.12880)

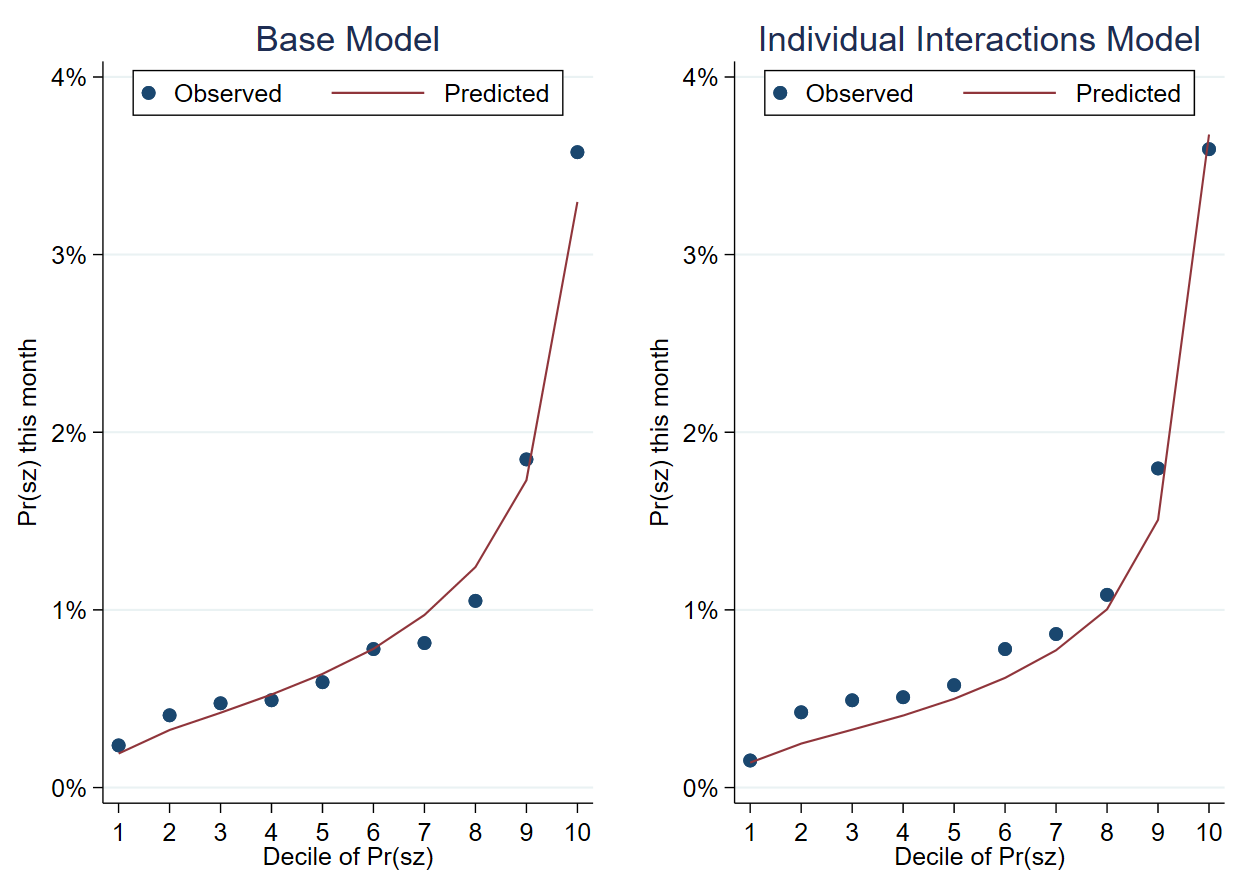

Supplement: Supplementary file 1 — Appendix S1. [file EPI4-9-333-s001.zip › supp fig 1.tif]

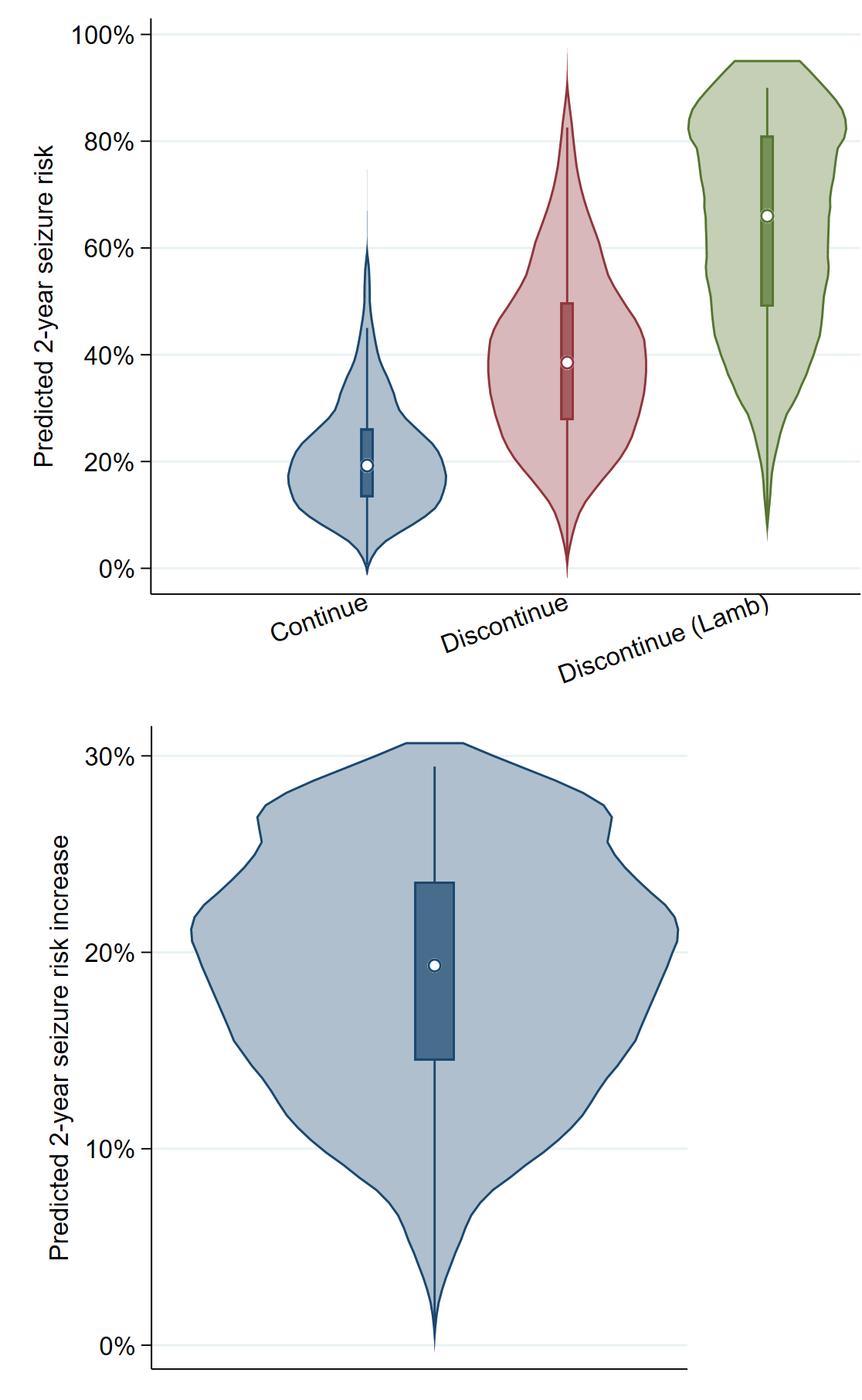

Supplement: Supplementary file 1 — Appendix S1. [file EPI4-9-333-s001.zip › supp fig 2.tif]

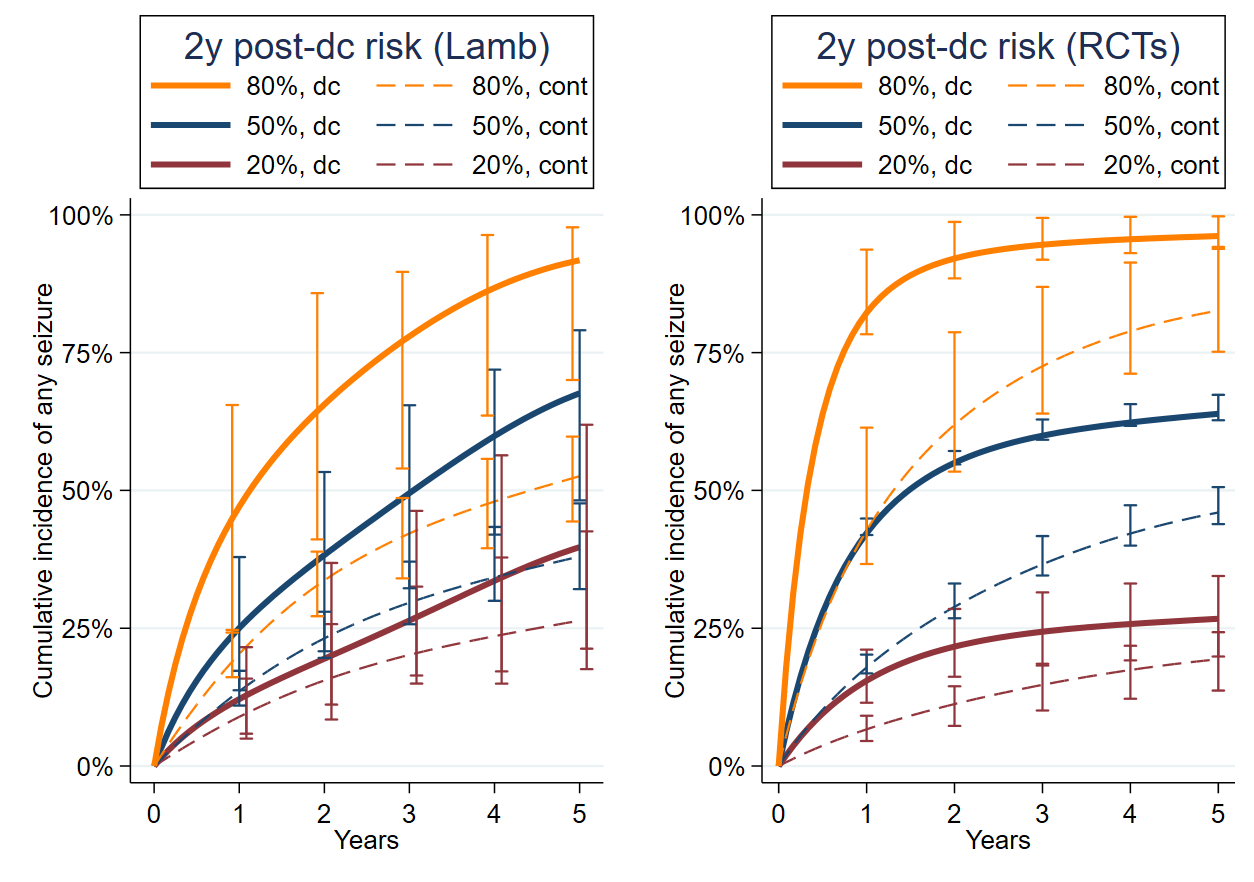

Supplement: Supplementary file 1 — Appendix S1. [file EPI4-9-333-s001.zip › supp fig 3.tif]

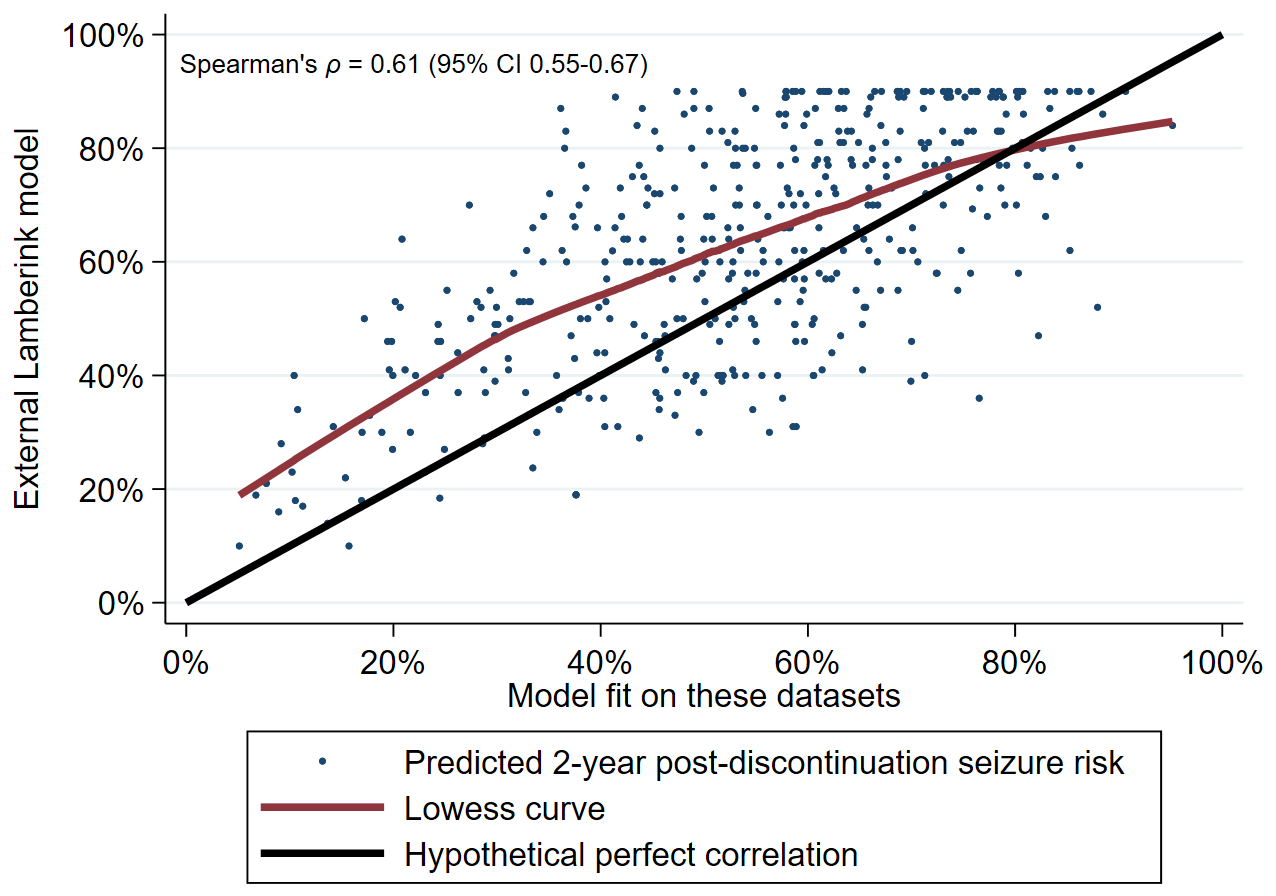

Supplement: Supplementary file 1 — Appendix S1. [file EPI4-9-333-s001.zip › supp fig 4.tif]

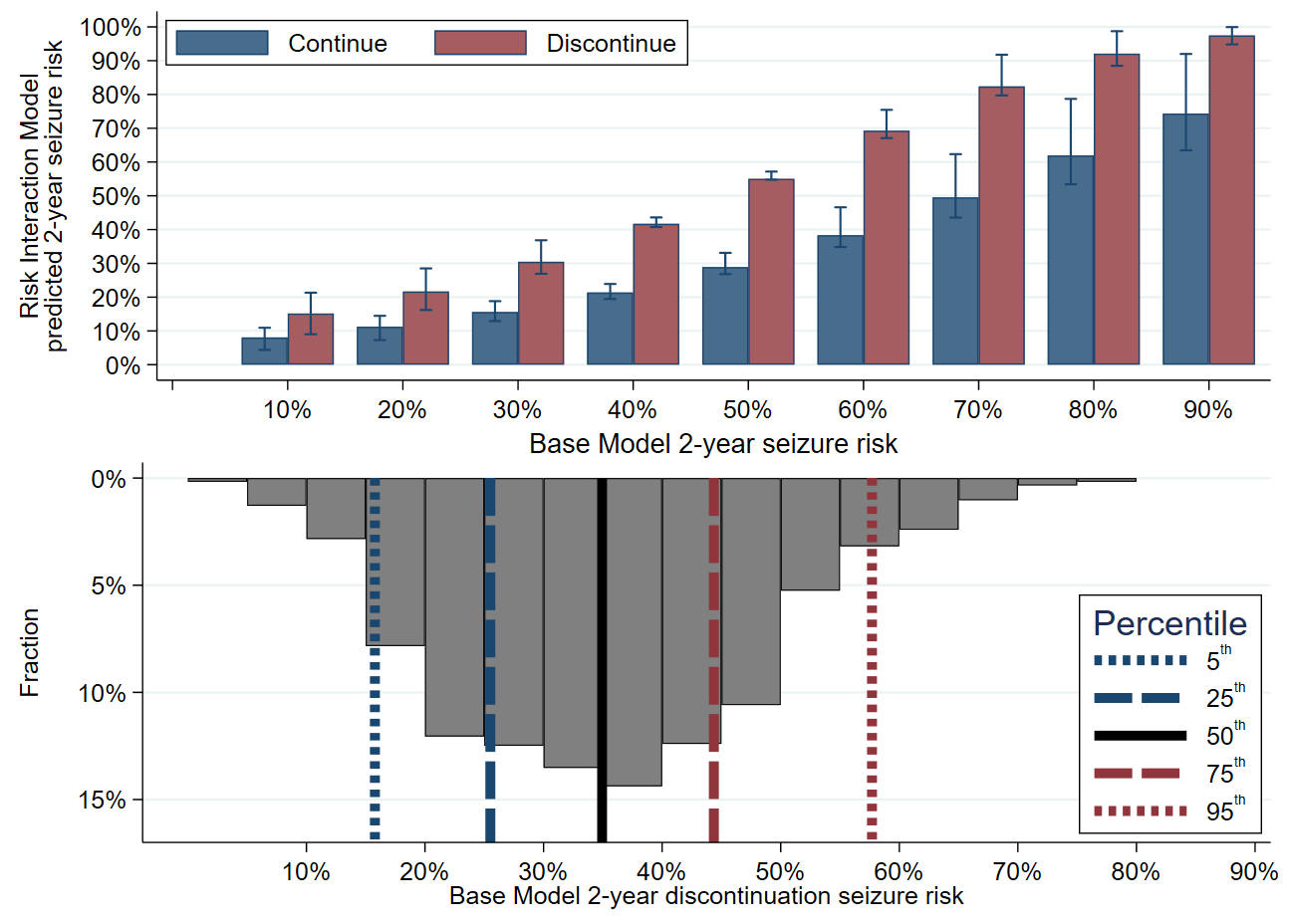

Supplement: Supplementary file 1 — Appendix S1. [file EPI4-9-333-s001.zip › supp fig 5.tif]
